# Procyanidin Protects Against Polystyrene Microplastic-Induced Male Reproductive Injury Through the NRF2/SLC7A11/GPX4 Pathway

**DOI:** 10.3390/ani16213382

**Published:** 2026-10-27

**Authors:** Shihui Zhang, Qiang Shan, Boshi Xu, Shuo Li, Zheng Cao, Yanan Li, Lijia Zhao

**Affiliations:** 1 College of Veterinary Medicine, Northeast Agricultural University, Harbin 150030, China; 2 Key Laboratory of the Provincial Education Department of Heilongjiang for Common Animal Disease Prevention and Treatment, Harbin 150030, China

**Keywords:** polystyrene microplastics, procyanidin, reproductive injury, ferroptosis, NRF2/SLC7A11/GPX4 pathway

## Abstract

Polystyrene microplastics (PS-MPs) are ubiquitous environmental contaminants of emerging concern owing to their potential adverse effects on male reproductive health. However, the mechanisms underlying PS-MP-induced reproductive injury remain poorly understood, and effective protective interventions are lacking. Here, we investigated the protective effects of procyanidin (PC), a naturally occurring polyphenolic antioxidant, against PS-MP-induced reproductive injury in mice and elucidated the underlying mechanisms using TM3 Leydig cells. In mice, PC treatment attenuated the PS-MP-induced reductions in serum testosterone levels, sperm concentration, survival, and motility. PC also reduced the incidence of sperm abnormalities and the severity of histopathological lesions in the testes and epididymis. PS-MP exposure increased testicular Fe^2+^ levels and lipid peroxidation while decreasing GSH levels and GSH-Px, CAT, and T-SOD activities; these changes were reversed by PC. PS-MP exposure also downregulated the expression of NRF2, SLC7A11, and GPX4 and altered the expression of ferroptosis-related markers; PC treatment largely reversed these changes. In TM3 cells, PC reduced PS-MP-induced Fe^2+^ accumulation and lipid peroxidation, preserved mitochondrial membrane potential, and attenuated mitochondrial permeability transition pore (MPTP) opening. These effects were weakened by Erastin, an inhibitor of system Xc^−^ that suppresses SLC7A11-mediated cystine uptake, and by ML385, an NRF2 inhibitor, suggesting the involvement of ferroptosis-related and NRF2-mediated signaling. Molecular docking predicted potential interactions between PC and NRF2 and SLC7A11. Collectively, these findings indicate that PC protects against PS-MP-induced male reproductive injury, potentially by modulating the NRF2/SLC7A11/GPX4 pathway.

## Simple Summary

Polystyrene microplastics are widespread environmental pollutants that may harm male fertility; however, how they damage the reproductive system and how this damage can be prevented are not fully understood. This study investigated whether procyanidin, a natural antioxidant found in some plants, can protect male mice and testicular cells from reproductive injury caused by polystyrene microplastics and examined the biological processes involved. In mice, exposure to these microplastics lowered testosterone levels and reduced sperm number, survival, and movement, while increasing sperm abnormalities and tissue damage. These effects were largely reversed by procyanidin treatment. Procyanidin also reduced harmful iron accumulation and oxidative damage and helped restore energy-producing structures in testicular cells. Blocking specific protective biological steps reduced the benefits of procyanidin, suggesting that these pathways may play an important role in its protective effects. Accordingly, procyanidin may help protect male reproductive health from damage caused by polystyrene microplastics by supporting the body’s natural antioxidant defenses and limiting iron-dependent cell damage. This study suggests a potential nutritional or therapeutic approach to reducing fertility risks associated with environmental pollution, although further studies are needed before human use.

## 1. Introduction

Microplastics (MPs), commonly defined as plastic particles smaller than 5 mm in diameter, have become widespread environmental contaminants of growing concern [1]. They are generated through the fragmentation and degradation of larger plastic products through physical, chemical, and biological processes and can be released directly from plastic-containing consumer and industrial products [2,3]. Their persistence and mobility allow MPs to travel through water, air, soil, and food chains, contributing to their widespread occurrence in marine and freshwater environments, agricultural soils, and even in remote regions [4,5]. Following exposure, MPs can enter the body and reach multiple organs, including the liver [6], kidney [7], lung [8], brain [9], cardiovascular system [10], and reproductive tissues [11]. Accumulating evidence indicates that MP exposure disturbs cellular homeostasis and induces oxidative stress, inflammation, apoptosis, and autophagy-related responses [12,13,14,15]. Given their widespread distribution and interactions with multiple biological systems, the potential health effects of MPs have attracted increasing attention.

Among the various types of MPs, polystyrene microplastics (PS-MPs) are widely used in consumer and industrial products, including disposable food containers, packaging materials, and thermal insulation [16]. Owing to their widespread use and environmental persistence, PS-MPs are used as a model to investigate the potential health effects of MP exposure. Growing evidence highlights the vulnerability of the male reproductive system to PS-MPs. Exposure to PS-MPs has been associated with impaired reproductive function in several aquatic species, including zebrafish [17], oysters [18], and marine medaka [19]. Similar effects have been reported in mammals, with PS-MP exposure associated with reduced sperm quality [20], impaired testosterone synthesis [21], disruption of the blood–testis barrier [22], and testicular inflammation [23]. These findings suggest that PS-MPs affect multiple aspects of male reproductive function, although the mechanisms underlying these effects remain poorly understood. Male reproductive dysfunction is an important contributor to infertility, and male factors are estimated to account for approximately half of infertility cases [24]. Therefore, elucidating the cellular and molecular mechanisms underlying PS-MP-induced reproductive injury is important for understanding the potential health risks associated with MP exposure.

Growing evidence indicates that oxidative stress contributes to PS-MP-induced reproductive injury, characterized by excessive reactive oxygen species (ROS) production and impaired antioxidant defenses. Persistent redox imbalance can promote lipid peroxidation and may contribute to ferroptosis, an iron-dependent form of regulated cell death distinct from apoptosis and necrosis [25]. Ferroptosis has been implicated in PS-MP-induced reproductive toxicity [26,27]. This form of regulated cell death is characterized by iron accumulation and excessive lipid peroxidation and has been linked to reproductive injury and impaired spermatogenesis [28]. A major cellular defense against ferroptosis is the SLC7A11/GPX4 system [29]. SLC7A11 mediates cysteine uptake in exchange for glutamate, providing the substrate for glutathione (GSH) synthesis, whereas GPX4 uses GSH to reduce phospholipid hydroperoxides and limit lipid peroxidation [30]. When this defense system is impaired, cysteine and GSH depletion, together with reduced GPX4 activity, can lead to the accumulation of lipid peroxides and increase susceptibility to ferroptotic cell death. These mechanisms suggest that ferroptosis, potentially involving dysfunction of the SLC7A11/GPX4 antioxidant defense system, may link PS-MP exposure to reproductive injury.

Nuclear factor erythroid 2-related factor 2 (NRF2) is a major regulator of cellular antioxidant defenses and plays an important role in maintaining redox homeostasis. Under basal conditions, NRF2 is continuously targeted for degradation through its interaction with Kelch-like ECH-associated protein 1 (KEAP1). Oxidative stress disrupts this regulatory process, allowing NRF2 to accumulate and translocate to the nucleus, where it activates the transcription of cytoprotective genes through antioxidant response elements. Among its downstream targets, NRF2 regulates genes involved in cellular protection against ferroptosis, including SLC7A11 and heme oxygenase-1 (HO-1) [31,32]. By promoting cystine uptake and maintaining GSH-dependent antioxidant capacity, NRF2-mediated regulation of SLC7A11 can help preserve GPX4 function and limit lipid peroxidation. The KEAP1/NRF2 pathway has also been implicated in the maintenance of testicular redox homeostasis and male reproductive functions under oxidative stress [33,34]. Nevertheless, whether impaired NRF2 signaling contributes to PS-MP-induced ferroptotic injury in the reproductive system and whether modulating the NRF2/SLC7A11/GPX4 axis can attenuate this injury remain unclear.

Procyanidin (PC) is a group of polyphenolic compounds widely distributed in fruits, nuts, and other plant-derived foods, including grapes, peanuts, apples, and blueberries [35]. PC has attracted considerable interest owing to its antioxidant and anti-inflammatory properties, as well as its reported protective effects against cardiovascular, immune, and other pathological conditions [36]. Grape seeds are an important source of PC, and previous studies have reported that PC exhibits strong free-radical-scavenging activity compared with conventional antioxidant compounds, such as vitamins C and E [37]. Given the potential roles of oxidative imbalance and ferroptosis in PS-MP-induced reproductive injury, the antioxidant properties of PC make it a potential candidate for mitigating MP-associated reproductive damage. PC-containing preparations are also available as dietary supplements [38], supporting further investigation of their biological effects and potential translational applications. However, whether PC can protect against PS-MP-induced male reproductive injury and the molecular mechanisms underlying any protective effects remain unclear.

In the present study, we investigated the protective effects of PC against PS-MP-induced male reproductive toxicity in mice and TM3 cells. We evaluated reproductive function, testicular injury, ferroptosis-related changes, and alterations in the NRF2/SLC7A11/GPX4 pathway. Erastin and ML385 treatments, along with molecular docking analysis, were used to further investigate the underlying molecular responses. These findings provide mechanistic insights into the potential protective effects of PC against PS-MP-induced reproductive injury.

## 2. Materials and Methods

### 2.1. Materials

PS-MPs (PS01050), 500 nm in diameter, were obtained from Ruige Biotechnology Co., Ltd. (Wuxi, China). PC (Y259379) and Erastin (SC0224) were obtained from Beyotime Biotechnology Co., Ltd. (Shanghai, China). ML385 (CM05324) was obtained from Proteintech Group, Inc. (Wuhan, China).

### 2.2. Animals and Treatment

Male KM mice aged 8 weeks and weighing 33 ± 3.5 g were obtained from Liaoning Changsheng Biotechnology Co., Ltd. (Benxi, China). Animals were housed under standard laboratory conditions at 22 ± 2 °C with a 12 h light/dark cycle and free access to food and water. All experimental procedures were reviewed and approved by the Animal Ethics Committee of Northeast Agricultural University (Approval No. NEAUEC20250346, approved on 10 March 2025, China). After acclimatization, mice were randomly assigned to four groups (n = 5 per group): Control, PS-MPs, PS-MPs + PC, and PC. PS-MPs were administered at 50 mg/kg/day, while PC was administered at 400 mg/kg/day. Both were dispersed in double-distilled water by sonication, and each animal received the corresponding suspension by oral gavage at a volume of 0.2 mL. Mice received the corresponding treatments by daily gavage for 4 consecutive weeks. Body weight was recorded weekly throughout the experimental period. At the end of the treatment period, necropsy and sample collection were performed between 9:00 a.m. and 11:00 a.m. on the following day. Mice were anesthetized with isoflurane and then euthanized by cervical dislocation. Blood, testes, and epididymides were rapidly collected. Testes and epididymides were weighed, and the corresponding organ coefficients were calculated. The PS-MP dose was selected based on previously reported estimates of human microplastic intake and mouse-equivalent exposure levels, whereas the PC dose was selected according to previously reported experimental studies [39,40].

### 2.3. Sperm Quality and Morphology Assessment

Each epididymis was minced and placed in pre-warmed physiological saline maintained at 37 °C. The tissue was incubated on a shaker for 10 min to allow sperm to disperse into the medium. 10 μL sperm suspension was placed on a microscope slide, and sperm quality was evaluated using computer-assisted sperm analysis (CASA). Sperm concentration, survival, and motility parameters, including average path velocity (VAP), straight-line velocity (VSL), and curve-line velocity (VCL), were assessed. For sperm morphology analysis, the sperm suspension was stained with eosin and examined microscopically. Sperm were classified according to their morphology, and normal and abnormal forms were quantified, including headless, tailless, and bent-midpiece sperm.

### 2.4. Hematoxylin and Eosin Staining

Testicular and epididymal tissues were collected and immediately fixed in 4% paraformaldehyde (Solarbio Technology Co., Ltd., Beijing, China) for 24 h at room temperature. The tissues were subsequently dehydrated through a graded ethanol series, embedded in paraffin, and sectioned using a microtome. Tissue sections were stained with hematoxylin and eosin (H&E), followed by dehydration, clearing, and mounting. Histopathological changes in the testis and epididymis were examined using an optical microscope.

### 2.5. Determination of Serum Testosterone

Serum testosterone concentrations were determined using a commercial ELISA kit (ml103518; Shanghai Enzyme-Linked Biotechnology Co., Ltd., Shanghai, China). The assay was performed according to the manufacturer’s instructions.

### 2.6. Determination of Ferrous Iron

Ferrous iron (Fe^2+^) levels in serum and testicular tissues were determined using an iron assay kit (BC5415; Solarbio Technology Co., Ltd., Beijing, China). Serum samples and testicular tissue homogenates were processed according to the manufacturer’s instructions, and Fe^2+^ concentrations were determined according to the manufacturer’s instructions.

### 2.7. Determination of Ferroptosis-Related Biochemical Parameters

To characterize ferroptosis-associated biochemical changes in the testis, lipid peroxide (LPO), malondialdehyde (MDA), reduced glutathione (GSH), glutathione peroxidase (GSH-Px), catalase (CAT), and total superoxide dismutase (T-SOD) were measured in testicular homogenates. LPO was measured using a commercial kit (BC5245; Solarbio Technology Co., Ltd., Beijing, China). MDA (A003-1), GSH (A006-1-1), GSH-Px (A005), CAT (A007-1), and T-SOD (A001-1) were measured using commercial kits from Nanjing Jiancheng Bioengineering Institute (Nanjing, China). All assays were performed according to the respective manufacturer’s instructions.

### 2.8. Immunofluorescence

Paraffin-embedded testicular tissue blocks were sectioned using a microtome. After dewaxing with xylene and rehydration through a graded ethanol series, the sections were subjected to heat-mediated antigen retrieval using antigen retrieval solution, followed by natural cooling, and were washed three times with PBS. The sections were blocked with 3% bovine serum albumin (BSA) for 30 min and incubated with primary antibody overnight at 4 °C. After being washed three times with PBS, the corresponding fluorescent secondary antibodies were added and incubated at room temperature in the dark for 50 min, followed by counterstaining with DAPI for 10 min. Finally, sections were incubated with autofluorescence quencher for 5 min, rinsed with water, and sealed with an anti-autofluorescence mounting medium. Images were collected under an inverted fluorescence microscope (Leica, Wetzlar, Germany).

### 2.9. CCK-8 Cell Viability Assay

The concentrations of PS-MPs and PC used for TM3 cell treatment were determined using a CCK-8 assay. Cells were exposed to different concentrations of PS-MPs or PC, and cell viability was evaluated using the CCK-8 assay (Beyotime Biotechnology Co., Ltd., Shanghai, China) according to the manufacturer’s protocol.

### 2.10. Cell Culture and Treatment

TM3 cells were cultured in high-glucose Dulbecco’s modified Eagle’s medium (DMEM, HyClone Laboratories, Inc., Logan, UT, USA) supplemented with 10% fetal bovine serum (FBS, ZETA life, Inc., Menlo Park, CA, USA) and 1% penicillin–streptomycin and maintained at 37 °C in a humidified incubator containing 5% CO_2_. Cultured TM3 cells were divided into the following groups: Control, PS-MPs, PS-MPs + PC, PS-MPs + PC + Erastin, and PS-MPs + PC + ML385. PS-MPs were used at 20 μg/mL and PC at 10 μg/mL. Erastin and ML385 were used at 3 μM and 5 μM, respectively [41]. Erastin or ML385 was added 2 h before the treatment. Cells were collected after 24 h of treatment for subsequent analyses.

### 2.11. Fluorescence Staining of Ferroptosis- and Mitochondria-Related Parameters

Several fluorescent probes were used to assess ferroptosis-associated and mitochondrial changes in TM3 cells. Intracellular Fe^2+^ was detected using FerroOrange (F374; Beiren Chemical Technology Co., Ltd., Beijing, China), and lipid peroxidation was assessed using BODIPY 581/591 C11 (S0043; Beyotime Biotechnology Co., Ltd., Shanghai, China). Mitochondrial membrane potential was evaluated using JC-1 (M8650; Solarbio Technology Co., Ltd., Beijing, China), while mitochondrial permeability transition pore (MPTP) opening was assessed using the MPTP assay kit (C2009S; Beyotime Biotechnology Co., Ltd., Shanghai, China). Following the indicated treatments, TM3 cells were incubated with the corresponding probes according to the manufacturer’s instructions. After staining, fluorescence images were acquired using an inverted fluorescence microscope under consistent imaging conditions, and fluorescence signals were quantified for comparison among experimental groups.

### 2.12. RNA Extraction and RT-qPCR

Total RNA was extracted from testicular tissues and TM3 cells using RNAiso Plus (Takara Bio Inc., Shiga, Japan) reagent. The extracted RNA was subjected to reverse transcription to cDNA using a reverse transcription kit (Takara Bio Inc., Shiga, Japan). RT-qPCR was subsequently performed using the cDNA as the template. The relative mRNA expression levels were determined. GAPDH was used as the internal reference gene, and relative gene expression was calculated using the 2^−ΔΔCt^ method. The primer sequences are provided in Supplementary Table S1.

### 2.13. Western Blot Analysis

Total proteins were extracted from testicular tissues and TM3 cells using RIPA lysis buffer (Beyotime Biotechnology Co., Ltd., Shanghai, China) supplemented with PMSF. Protein concentrations were determined using a BCA protein assay kit (Beyotime Biotechnology Co., Ltd., Shanghai, China). Equal amounts of protein were separated by means of SDS-PAGE and subsequently transferred onto PVDF membranes. The membranes were blocked with protein-free rapid blocking buffer for 15 min and incubated overnight at 4 °C with the primary antibodies. After washing, the membranes were incubated with HRP-conjugated secondary antibodies at room temperature for 60 min. Protein bands were visualized using an enhanced chemiluminescence (ECL, Beyotime Biotechnology Co., Ltd., Shanghai, China) reagent and captured using a chemiluminescence imaging system (Shanghai Tanon Life Science Co., Ltd., Shanghai, China). The sources and dilution ratios of the antibodies used for detection are provided in Supplementary Table S2.

### 2.14. Molecular Docking Analysis

The three-dimensional structures of NRF2 and SLC7A11 were retrieved from the UniProt database (https://www.uniprot.org/), while the two-dimensional chemical structure of PC was obtained from the ChemSpider database (https://www.chemspider.com/). The two-dimensional structure of PC was converted into its corresponding three-dimensional structure using Chem3D 23.1.1.3 software. Molecular docking was performed using AutoDock Vina 1.2.5 to evaluate the potential interactions of PC with NRF2 and SLC7A11. The resulting docking complexes were analyzed and visualized using PyMOL 3.1.8 software. The predicted binding energies and interaction modes were used to assess the potential binding affinity of PC toward NRF2 and SLC7A11.

### 2.15. Statistical Analysis

Data are presented as mean ± standard deviation (SD). Statistical analyses were performed using GraphPad Prism 10.0 software. Differences among multiple experimental groups were evaluated using One-way analysis of variance followed by an appropriate post hoc test. A value of *p* < 0.05 was considered statistically significant.

## 3. Results

### 3.1. Effect of PC on PS-MP-Induced Male Reproductive Dysfunction and Reproductive Tissue Injury

**Figure 1:**
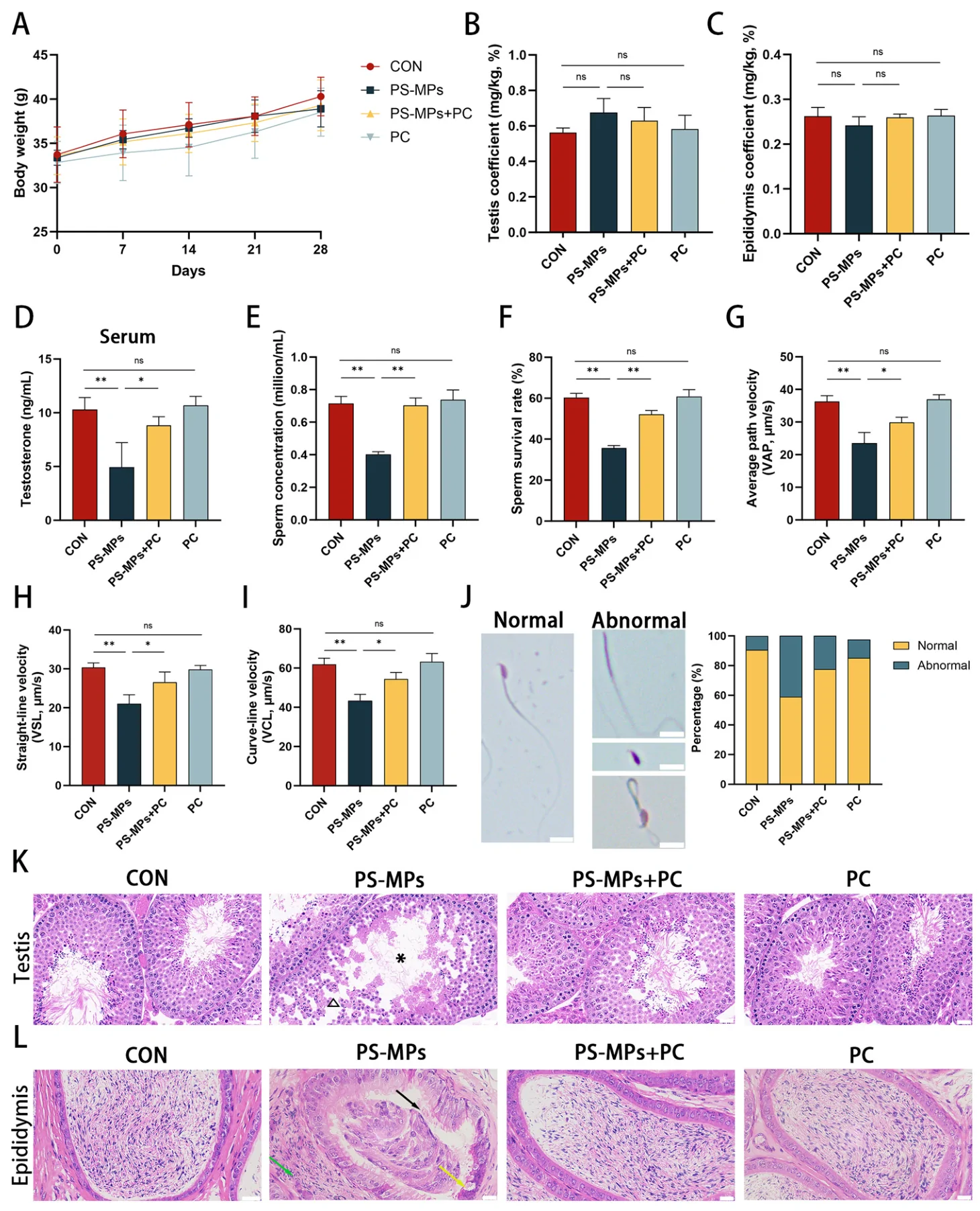
Effect of PC on PS-MP-induced male reproductive dysfunction and tissue injury in mice. (**A**) Body weight changes during the 4-week treatment period. (**B**) Testis coefficient and (**C**) epididymis coefficient. (**D**) Serum testosterone concentration. (**E**) Sperm concentration and (**F**) sperm survival rate. (**G**–**I**) Sperm motility parameters, including average path velocity (VAP), straight-line velocity (VSL), and curve-line velocity (VCL). (**J**) Representative images of sperm morphology and quantitative analysis of abnormal sperm, including headless, tailless, and bent-midpiece spermatozoa. Scale bar, 10 μm. (**K**) Representative H&E-stained testicular sections. Asterisks indicate enlarged seminiferous tubular lumens, and triangles indicate loss and disorganization of germ cells. Scale bar, 25 μm. (**L**) Representative H&E-stained epididymal sections. Black arrows indicate epithelial cell shedding, yellow arrows indicate epithelial cell necrosis, and green arrows indicate disruption of the epididymal duct structure. Scale bar, 25 μm. Data are presented as mean ± SD. * *p* < 0.05, ** *p* < 0.01; ns, not significant.

To evaluate the protective effect of PC against PS-MP-induced reproductive toxicity, mice were divided into four groups: Control, PS-MPs, PS-MPs + PC, and PC. After 4 weeks of treatment, no significant differences in body weight were observed among the groups. Similarly, neither the testis coefficient nor the epididymis coefficient differed significantly among the groups (*p* > 0.05) (Figure 1A–C). PS-MP exposure significantly decreased serum testosterone levels compared with the Control group (*p* < 0.01), whereas PC supplementation markedly attenuated this decrease (*p* < 0.05) (Figure 1D). Sperm analysis showed that PS-MP exposure significantly reduced sperm concentration and survival (*p* < 0.01), while PC treatment significantly improved both parameters (*p* < 0.01) (Figure 1E,F). In addition, sperm motility parameters, including average path velocity (VAP), straight-line velocity (VSL), and curve-line velocity (VCL), significantly decreased following PS-MP exposure and were significantly restored by PC treatment (*p* < 0.05) (Figure 1G–I). Sperm morphological analysis further showed that PS-MP exposure increased the proportion of abnormal sperm, including headless, tailless, and bent-midpiece sperm, accompanied by a decrease in the proportion of normal sperm. PC treatment markedly increased the proportion of morphologically normal sperm (*p* < 0.05) (Figure 1J). Histopathological examination revealed obvious structural alterations in the testis and epididymis following PS-MP exposure. In the testis, PS-MP exposure was associated with enlarged seminiferous tubular lumens, thinning of the spermatogenic epithelium, reduced sperm content, and disordered germ-cell arrangement. These changes were markedly alleviated by PC treatment, with more compact germ-cell organization and increased sperm content within the tubular lumens (Figure 1K). In the epididymis, PS-MP exposure caused disruption of the epididymal tubular structure, epithelial cell necrosis and shedding, and reduced sperm content. These pathological changes were substantially less pronounced following PC treatment (Figure 1L).

### 3.2. Effect of PC on PS-MP-Induced Iron Accumulation and Oxidative Imbalance

**Figure 2:**
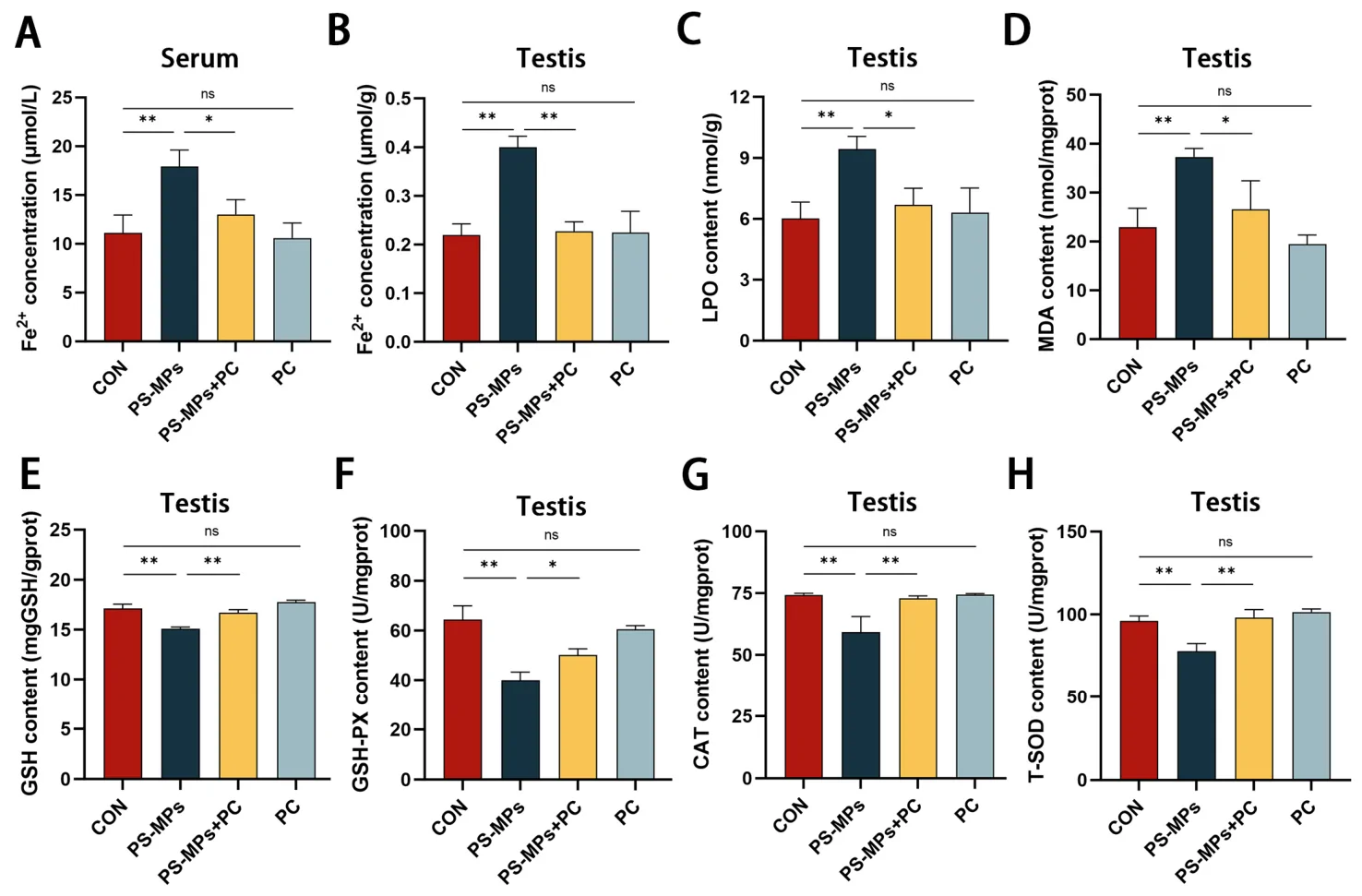
Effect of PC on PS-MP-induced iron accumulation and oxidative imbalance in the testis. (**A**) Serum Fe^2+^ concentration. (**B**) Testicular Fe^2+^ concentration. (**C**) Testicular LPO level. (**D**) Testicular MDA content. (**E**) Testicular GSH content. (**F**) GSH-Px activity. (**G**) CAT activity. (**H**) T-SOD activity. Data are presented as mean ± SD. * *p* < 0.05, ** *p* < 0.01; ns, not significant.

To determine whether PS-MP exposure altered iron homeostasis and redox status, Fe^2+^ levels and several indicators of lipid peroxidation and antioxidant capacity were examined. PS-MP exposure significantly increased Fe^2+^ levels in both serum and testicular tissue (*p* < 0.01), whereas PC treatment markedly attenuated these increases (*p* < 0.05) (Figure 2A,B). PS-MP exposure also significantly increased the levels of LPO and MDA in testicular tissue (*p* < 0.01). PC supplementation significantly reduced both parameters compared with the PS-MPs group (*p* < 0.05) (Figure 2C,D). In parallel, PS-MP exposure decreased GSH content and the activities of GSH-Px, T-SOD, and CAT (*p* < 0.01). These changes were significantly alleviated by PC treatment, with antioxidant parameters restored toward the Control levels (*p* < 0.05) (Figure 2E–H). Thus, PS-MP exposure was associated with increased Fe^2+^ accumulation and lipid peroxidation together with reduced antioxidant capacity in the testis, whereas PC treatment substantially attenuated these alterations.

### 3.3. Effect of PC on PS-MP-Induced Changes in Ferroptosis-Related Signaling in the Testis

**Figure 3:**
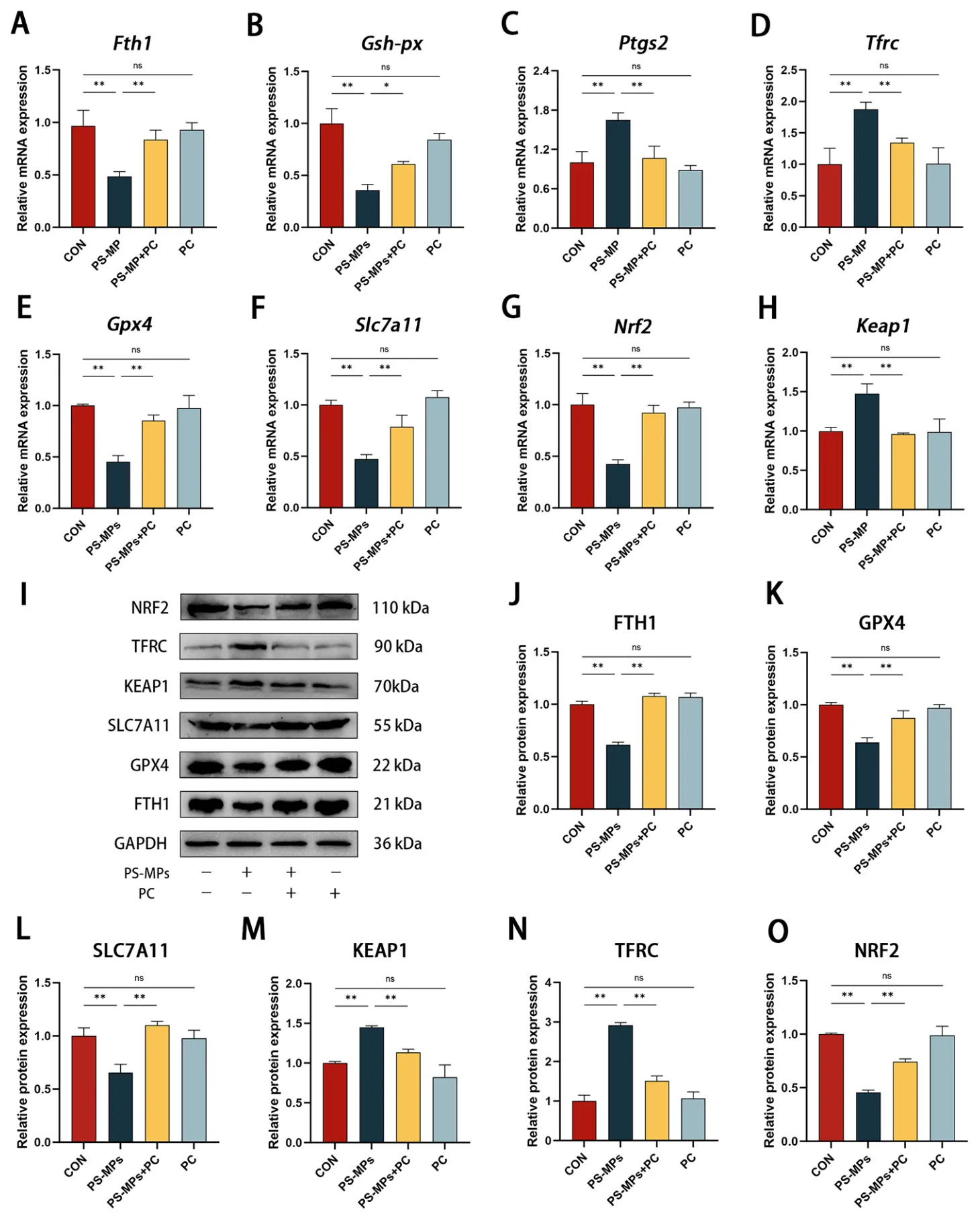
Effect of PC on PS-MP-induced changes in ferroptosis-related signaling in the testis. (**A**–**H**) Relative mRNA expression levels of *Fth1*, *Gsh-px*, *Ptgs2*, *Tfrc*, *Gpx4*, *Slc7a11*, *Nrf2*, and *Keap1* in testicular tissue. (**I**) Representative Western blot bands for NRF2, TFRC, KEAP1, SLC7A11, GPX4, FTH1, and GAPDH. (**J**–**O**) Quantitative analysis of the relative protein expression levels. Data are presented as mean ± SD. * *p* < 0.05, ** *p* < 0.01; ns, not significant.

**Figure 4:**
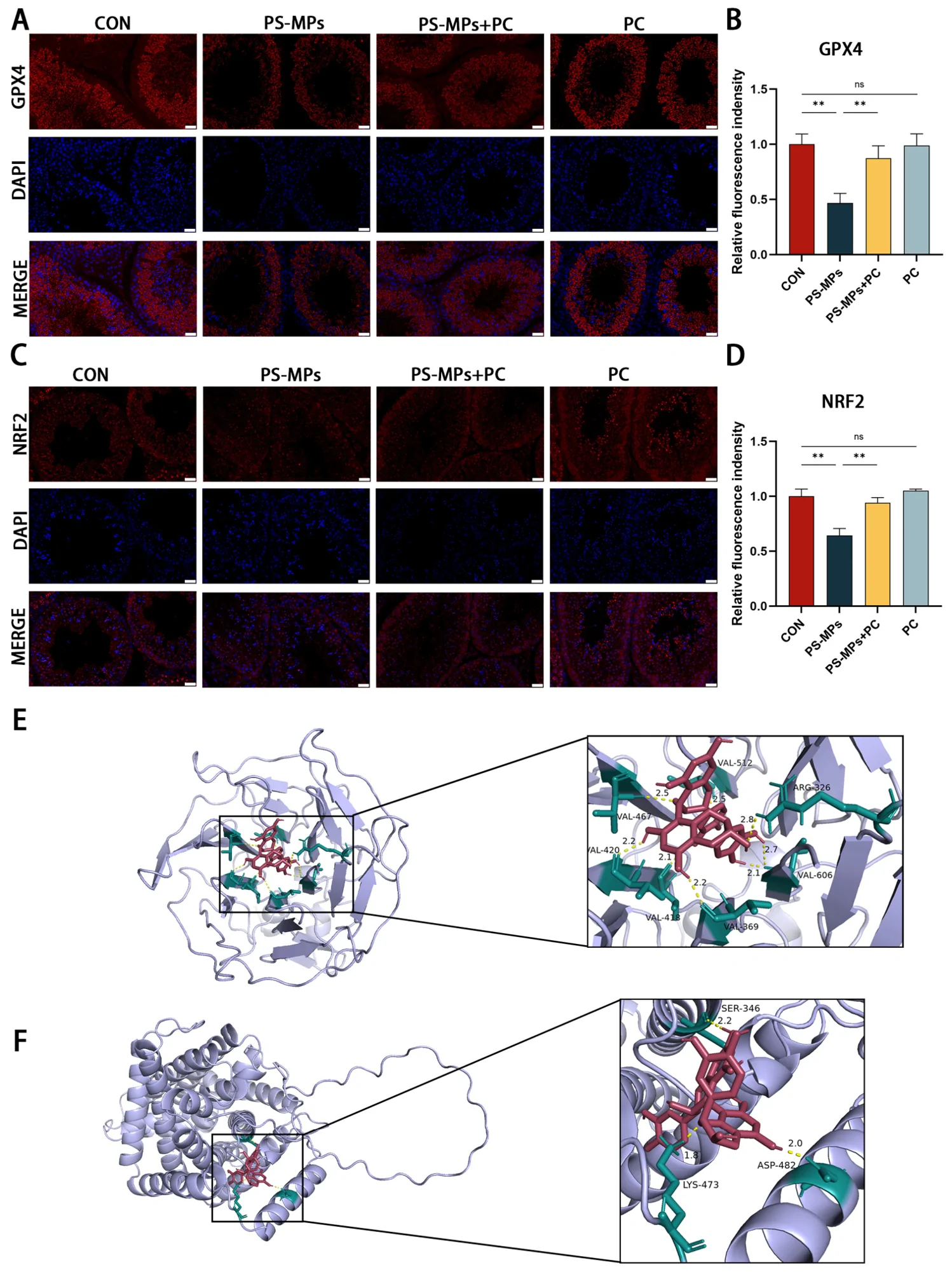
Effect of PC on NRF2 and GPX4 expression and predicted interactions of PC with NRF2 and SLC7A11. (**A**,**C**) Representative immunofluorescence images showing NRF2 and GPX4 expression (red) in testicular sections. Nuclei were counterstained with DAPI (blue). Scale bars, 50 μm. (**B**,**D**) Quantitative analysis of the relative fluorescence intensity of NRF2 and GPX4, respectively. (**E**) Molecular docking model showing the predicted interaction between PC and NRF2. (**F**) Molecular docking model showing the predicted interaction between PC and SLC7A11. Data are presented as mean ± SD. ** *p* < 0.01; ns, not significant.

To further examine the changes associated with altered iron and redox status, the expression of ferroptosis-related genes was analyzed by means of RT-qPCR. Compared with the Control group, PS-MP exposure significantly decreased the mRNA levels of *Fth1*, *Gsh-px*, *Gpx4,* and *Slc7a11*, while increasing the expression of *Ptgs2* and *Tfrc* (*p* < 0.01). In addition, *Nrf2* expression was decreased and *Keap1* expression was increased following PS-MP exposure (*p* < 0.01). PC treatment markedly attenuated these PS-MP-induced changes in mRNA levels (*p* < 0.05) (Figure 3A–H). Western blot analysis showed a similar pattern at the protein level. PS-MP exposure decreased the expression of FTH1, GPX4, SLC7A11, and NRF2, while increasing KEAP1 and TFRC expression (*p* < 0.01). PC supplementation substantially reversed these changes (*p* < 0.01) (Figure 3I–O and Figure S1). Immunofluorescence analysis further showed that PS-MP exposure reduced NRF2 and GPX4 fluorescence signals in testicular tissue (*p* < 0.01). PC treatment markedly increased the fluorescence signals of both proteins compared with the PS-MPs group (*p* < 0.01) (Figure 4A–D). Together, these results show that PS-MP exposure was accompanied by changes in ferroptosis-related markers and suppression of the NRF2/SLC7A11/GPX4-associated antioxidant defense, whereas PC treatment largely restored this molecular response.

### 3.4. Predicted Interactions of PC with NRF2 and SLC7A11

To further explore the potential molecular basis of the effects of PC on the NRF2/SLC7A11/GPX4-related pathway, molecular docking analysis was performed with NRF2 and SLC7A11. PC showed predicted binding energies of −10.1 kcal/mol for NRF2 and −8.6 kcal/mol for SLC7A11 (Figure 4E,F).

### 3.5. Effect of PC on PS-MP-Induced Iron Accumulation and Redox Imbalance in TM3 Cells

**Figure 5:**
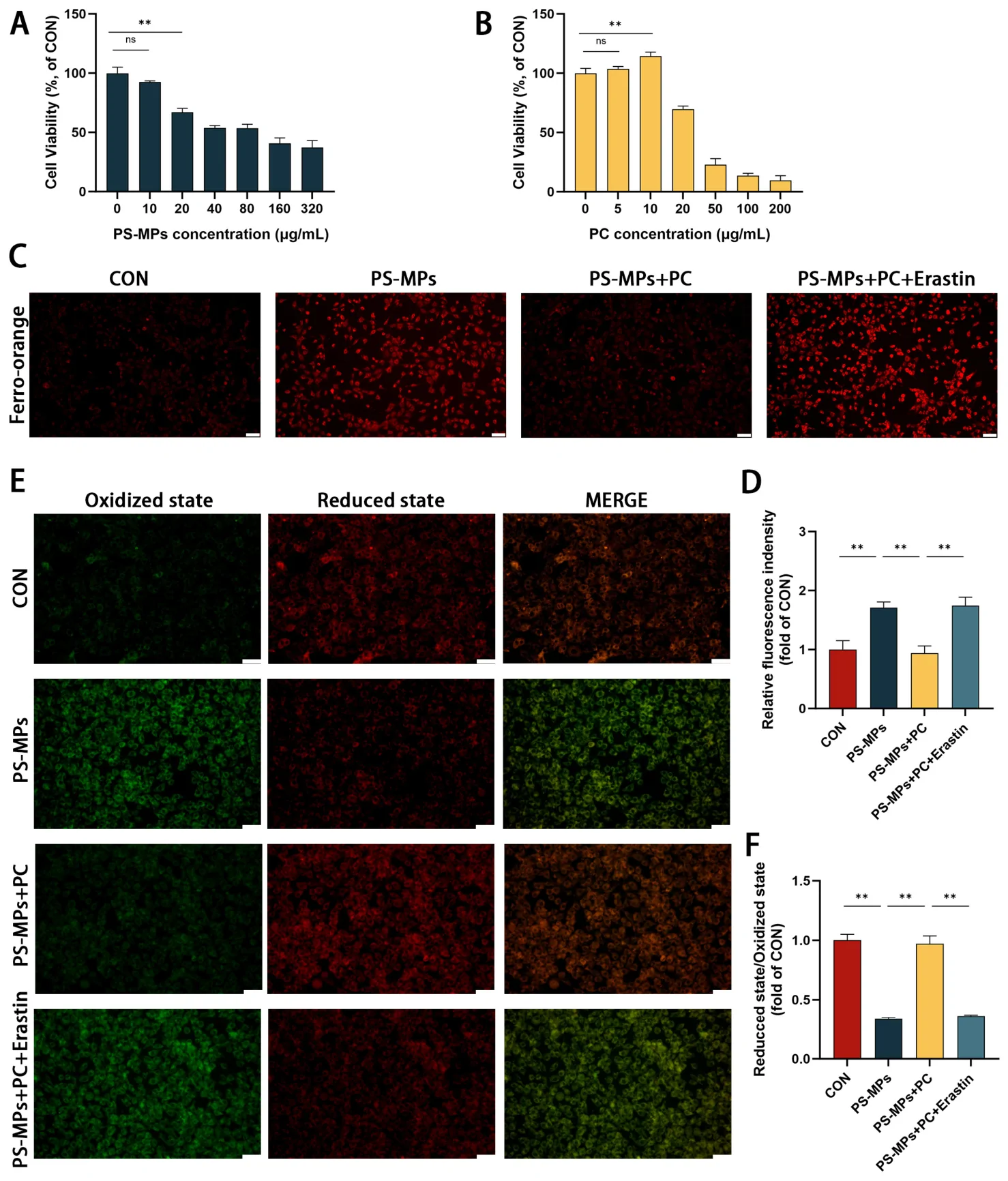
Effect of PC on PS-MP-induced iron accumulation and lipid peroxidation in TM3 cells. (**A**) Cell viability of TM3 cells following exposure to different concentrations of PS-MPs (0–320 μg/mL) for 24 h. (**B**) Cell viability of TM3 cells following exposure to different concentrations of PC (0–200 μg/mL) for 24 h. (**C**) Representative fluorescence images of intracellular Fe^2+^ detected using FerroOrange staining (red). Scale bars, 50 μm. (**D**) Quantitative analysis of FerroOrange fluorescence intensity. (**E**) Representative fluorescence images showing lipid peroxidation detected using C11-BODIPY staining (oxidized state: green; reduced state: red). Scale bars, 50 μm. (**F**) Quantitative analysis of the corresponding fluorescence signal. Data are presented as mean ± SD. ** *p* < 0.01; ns, not significant.

To further investigate the cellular response to PS-MP exposure and PC treatment, TM3 cells were used as an in vitro model. CCK-8 assays were first performed to determine suitable concentrations of PS-MPs and PC. Based on the results, 20 μg/mL PS-MPs and 10 μg/mL PC were selected for subsequent experiments (Figure 5A,B). FerroOrange staining showed that PS-MP exposure markedly increased intracellular Fe^2+^ levels in TM3 cells (*p* < 0.01). PC treatment significantly reduced this increase, whereas the addition of Erastin substantially weakened the reduction in intracellular Fe^2+^ produced by PC (*p* < 0.01) (Figure 5C,D). Assessment of cellular redox status further showed that PS-MP exposure increased oxidized signals and decreased reduced signals, indicating an altered cellular redox state (*p* < 0.01). PC treatment significantly alleviated these changes, whereas Erastin diminished the protective effect of PC (*p* < 0.01) (Figure 5E,F). These results indicate that PC attenuated PS-MP-associated iron accumulation and redox imbalance in TM3 cells, whereas pharmacological induction of ferroptosis with Erastin weakened these effects.

### 3.6. Effect of PC on PS-MP-Induced Mitochondrial Dysfunction in TM3 Cells

**Figure 6:**
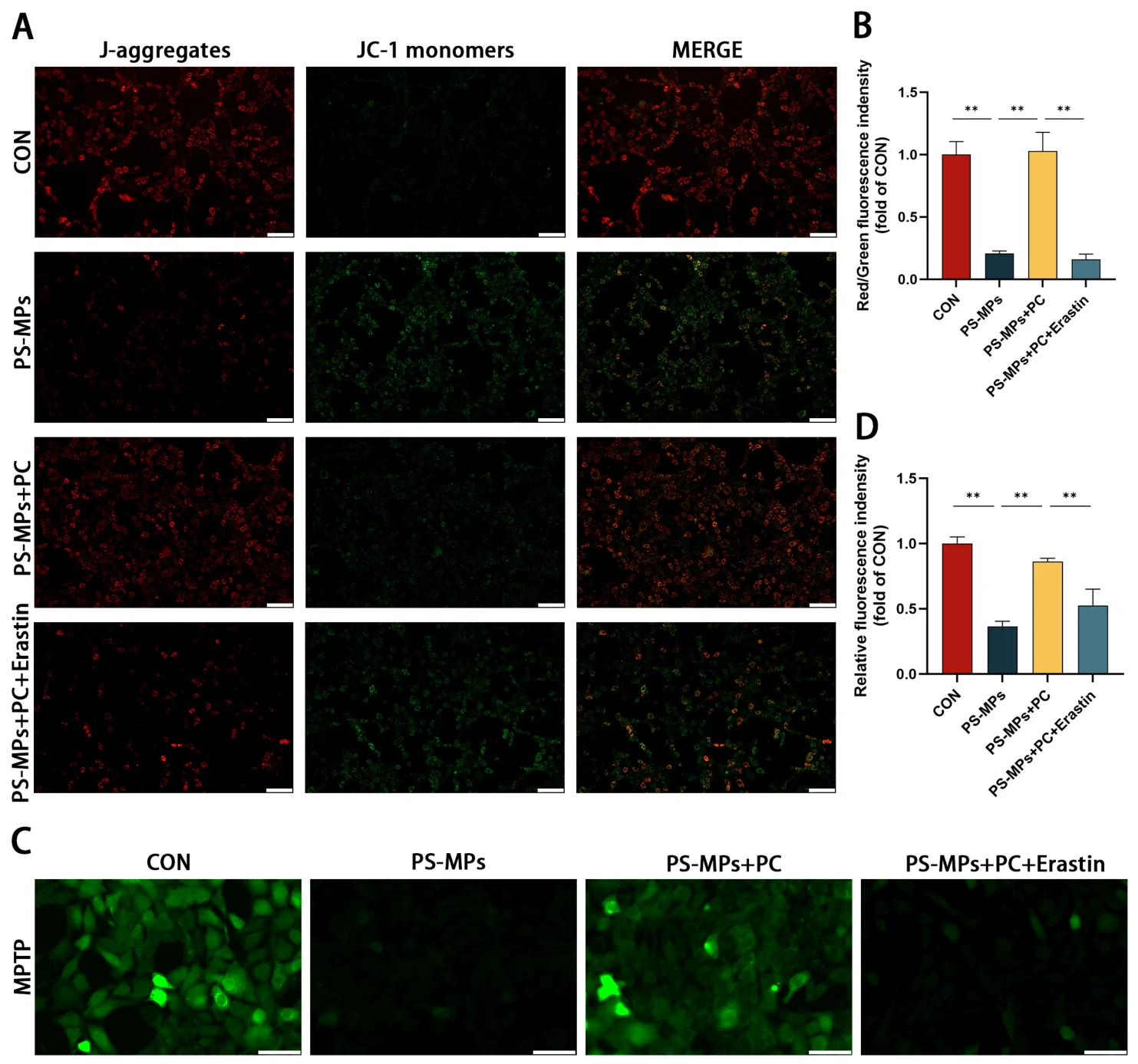
Effect of PC on PS-MP-induced mitochondrial dysfunction in TM3 cells. (**A**) Representative JC-1 fluorescence images showing mitochondrial membrane potential in TM3 cells. Red fluorescence represents J-aggregates in polarized mitochondria, whereas green fluorescence represents JC-1 monomers in depolarized mitochondria. Scale bar, 50 μm. (**B**) Quantitative analysis of the red-to-green fluorescence intensity ratio (J-aggregates/JC-1 monomers). (**C**) Representative fluorescence images showing MPTP opening in TM3 cells. Scale bar, 50 μm. (**D**) Quantitative analysis of relative MPTP fluorescence intensity. Data are presented as mean ± SD. ** *p* < 0.01.

Because mitochondrial function can be affected by cellular oxidative stress, mitochondrial membrane potential and mitochondrial permeability transition pore (MPTP) opening were subsequently examined. JC-1 staining showed that PS-MP exposure significantly decreased mitochondrial membrane potential in TM3 cells (*p* < 0.01). PC treatment markedly attenuated this decrease, whereas the protective effect of PC was weakened by Erastin treatment (*p* < 0.01) (Figure 6A,B). Consistent with the changes in mitochondrial membrane potential, PS-MP exposure increased MPTP opening, whereas PC treatment significantly reduced MPTP opening (*p* < 0.01). Addition of Erastin attenuated the effect of PC and resulted in greater MPTP opening compared with the PS-MPs + PC group (*p* < 0.01) (Figure 6C,D). Thus, PC treatment was associated with preservation of mitochondrial membrane potential and reduced MPTP opening in PS-MP-exposed TM3 cells, while Erastin weakened these effects.

### 3.7. Effect of SLC7A11 Inhibition on the Protective Effects of PC Against PS-MP-Induced Ferroptosis-Related Signaling

**Figure 7:**
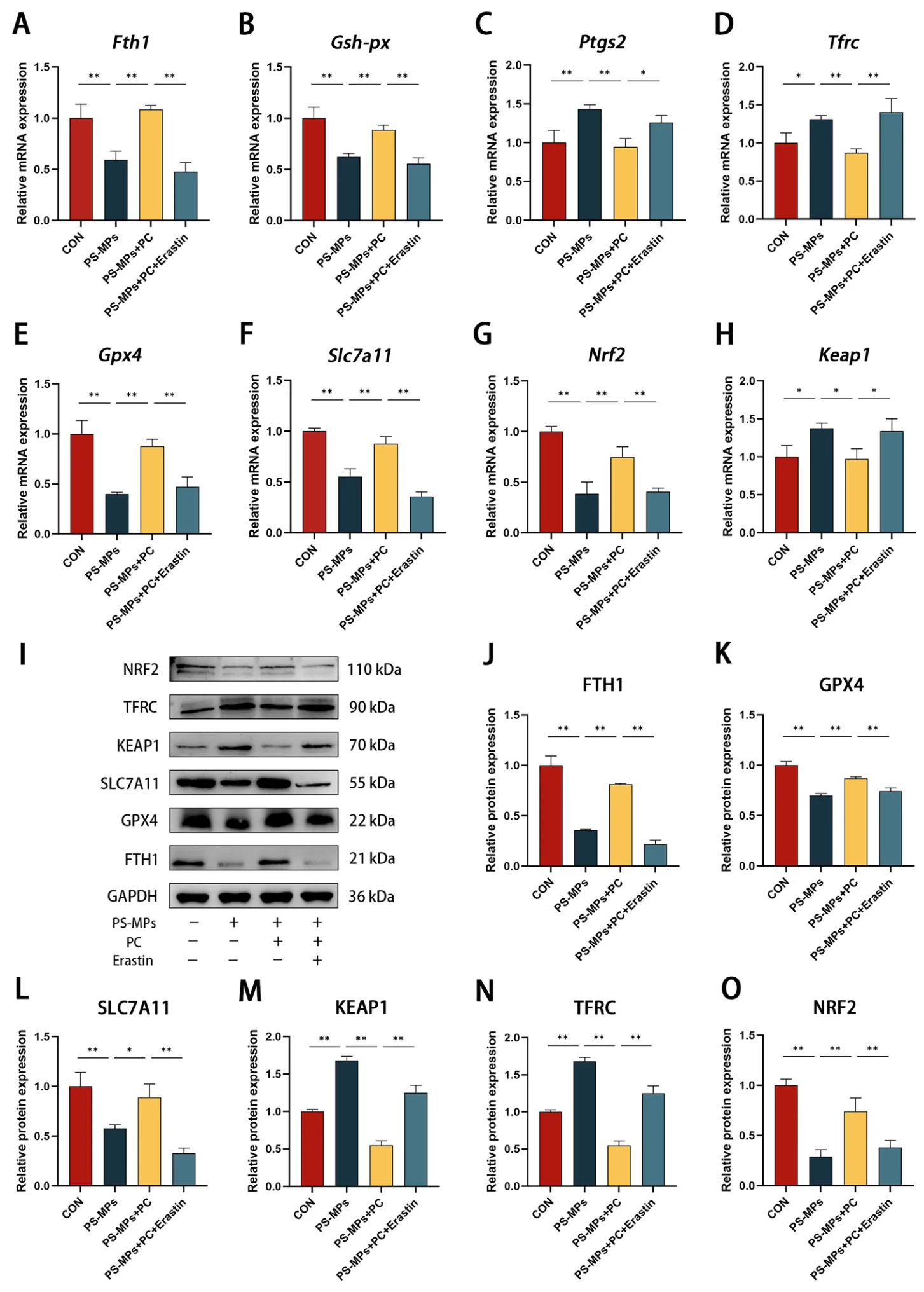
Effect of SLC7A11 inhibition on the PC-mediated response to PS-MP exposure in TM3 cells. (**A**–**H**) Relative mRNA expression levels of *Fth1*, *Gsh-px*, *Ptgs2*, *Tfrc*, *Gpx4*, *Slc7a11*, *Nrf2*, and *Keap1*, respectively. (**I**) Representative Western blot images of NRF2, TFRC, KEAP1, SLC7A11, GPX4, FTH1, and GAPDH. (**J**–**O**) Quantitative analysis of the relative protein expression levels. Data are presented as mean ± SD. * *p* < 0.05, ** *p* < 0.01.

To further examine the involvement of the SLC7A11/GPX4-associated defense system, the expression of ferroptosis-related genes and proteins was analyzed following Erastin treatment. PS-MP exposure significantly decreased the mRNA levels of *Gpx4*, *Slc7a11*, *Fth1*, and *Gsh-px*, while increasing *Ptgs2* and *Tfrc* expression. *Nrf2* expression was decreased and *Keap1* expression was increased (*p* < 0.05) (Figure 7A–H). PC treatment substantially attenuated these PS-MP-induced changes in mRNA expression (*p* < 0.05). However, addition of Erastin markedly weakened the effects of PC, with the expression profiles of ferroptosis-related genes shifting toward those observed in the PS-MPs group (*p* < 0.05) (Figure 7A–H). Western blot analysis showed corresponding changes at the protein level. PC treatment restored the expression of ferroptosis- and antioxidant-related proteins altered by PS-MP exposure, whereas Erastin substantially attenuated these PC-associated changes (*p* < 0.05) (Figure 7I–O and Figure S2). These findings indicate that pharmacological interference with SLC7A11-associated ferroptosis signaling weakens the protective effects of PC and is accompanied by loss of the PC-associated regulation of the SLC7A11/GPX4-related response.

### 3.8. Effect of NRF2 Inhibition on the PC-Mediated Regulation of the SLC7A11/GPX4 Pathway

**Figure 8:**
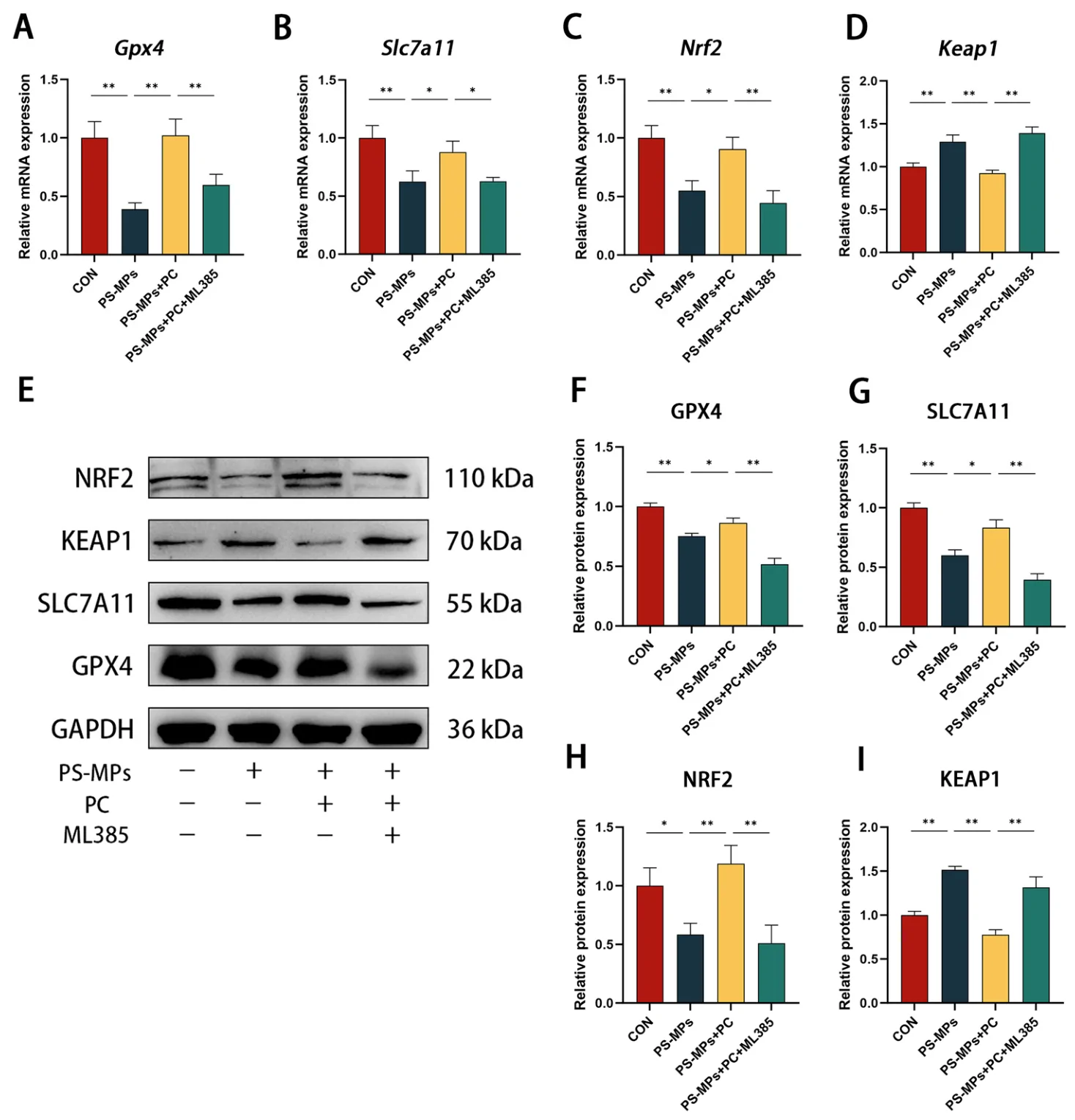
Effect of NRF2 inhibition on the PC-mediated regulation of the SLC7A11/GPX4 pathway in TM3 cells. (**A**–**D**) Relative mRNA expression levels of *Gpx4, Slc7a11, Nrf2*, and *Keap1*, respectively. (**E**) Representative Western blot images of NRF2, KEAP1, SLC7A11, GPX4, and GAPDH. (**F**–**I**) Quantitative analysis of the relative protein expression levels. Data are presented as mean ± SD. * *p* < 0.05, ** *p* < 0.01.

Because PC restored both NRF2 and the downstream SLC7A11/GPX4 pathway following PS-MP exposure, we next examined whether NRF2 is required for the PC-mediated regulation of SLC7A11/GPX4. To further investigate the contribution of NRF2 signaling to the effects of PC, TM3 cells were treated with the NRF2 inhibitor ML385 in the presence of PS-MPs and PC. PS-MP exposure significantly reduced the mRNA and protein expression of GPX4, SLC7A11, and NRF2, while increasing KEAP1 expression (*p* < 0.05). PC treatment markedly reversed these changes (*p* < 0.05) (Figure 8). However, the presence of ML385 substantially attenuated the effects of PC, markedly reducing the PC-induced increases in NRF2, SLC7A11, and GPX4, while weakening the PC-mediated suppression of KEAP1 (*p* < 0.01) (Figure 8A–I and Figure S3). These results indicate that inhibition of NRF2 signaling weakens the PC-associated regulation of the SLC7A11/GPX4 pathway in PS-MP-exposed TM3 cells.

## 4. Discussion

Previous studies have shown that PS-MP exposure can impair male reproductive function, including testosterone production, sperm quality, and testicular integrity [42]. However, the mechanisms underlying PS-MP-induced reproductive injury remain poorly understood, and effective interventions have yet to be established. In the present study, we investigated the protective effects of PC against PS-MP-induced male reproductive injury and examined the involvement of ferroptosis and the NRF2/SLC7A11/GPX4 pathway in mediating these effects. In mice, PS-MP exposure impaired reproductive function and was accompanied by iron accumulation, lipid peroxidation, depletion of antioxidant defenses, and suppression of the SLC7A11/GPX4 pathway, whereas PC substantially alleviated these alterations. Complementary experiments in TM3 cells, together with pharmacological interventions using Erastin and ML385, further supported the involvement of SLC7A11- and NRF2-related signaling in the protective effects of PC. Molecular docking provided additional evidence for potential interactions between PC and NRF2 and between PC and SLC7A11. Collectively, these findings suggest that PC protects against PS-MP-induced male reproductive injury and that modulation of the NRF2/SLC7A11/GPX4-associated antioxidant defense against ferroptosis may contribute to this protective effect.

Male reproductive function depends on the coordinated activity of Leydig cells, Sertoli cells, germ cells, and the epididymal environment. Previous studies have reported that PS-MP exposure disrupts sperm quality, testosterone production, and reproductive tissue integrity [42]. Consistent with these observations, the present study demonstrated that PS-MP exposure substantially reduced serum testosterone levels, sperm concentration, and sperm motility while increasing sperm abnormalities and inducing histopathological changes in the testes and epididymis. The simultaneous impairment of hormonal, functional, and structural endpoints suggests that PS-MPs affect multiple aspects of the male reproductive system, rather than a single reproductive parameter. Testosterone is particularly important in this context because adequate androgen signaling is required to maintain the testicular microenvironment and support normal spermatogenesis [43]. Therefore, the PS-MP-induced reduction in testosterone levels may be associated with the observed decline in sperm production and quality. The histopathological findings further support this interpretation, as disruption of seminiferous tubule architecture and epididymal structure may contribute to the observed alterations in sperm parameters.

PC administration substantially alleviated PS-MP-induced reproductive impairment. PC restored serum testosterone levels and improved sperm concentration, viability, and motility, while reducing sperm abnormalities and ameliorating testicular and epididymal lesions. The improvement across multiple reproductive parameters suggests that the protective action of PC extends beyond changes in a single functional outcome. Given the close relationship between oxidative stress and reproductive dysfunction, the antioxidant properties of PC may partly explain its beneficial effects [44,45]. However, its direct antioxidant activity alone may not fully explain the coordinated improvements in reproductive function observed in this study. Therefore, we examined whether PC modulates ferroptosis-related oxidative injury, which may represent an important cellular process linking oxidative stress to testicular dysfunction.

Ferroptosis is an iron-dependent form of regulated cell death characterized by the accumulation of redox-active iron and lipid peroxidation, together with weakened antioxidant defenses [46]. The findings of the present study were consistent with features associated with ferroptosis. PS-MP exposure increased Fe^2+^ concentrations in serum and testicular tissue and was accompanied by elevated LPO and MDA levels. Simultaneously, GSH, GSH-Px, T-SOD, and CAT levels were reduced, indicating substantial disruption of the antioxidant system. Iron accumulation is particularly relevant because redox-active iron can catalyze oxidative reactions and promote the formation of ROS species, thereby increasing the susceptibility of membrane lipids to peroxidation [28]. The increase in lipid peroxidation and depletion of antioxidant defenses suggest a biochemical link between iron dysregulation and cellular injury following PS-MP exposure. PC treatment substantially reversed these changes, suggesting that its protective effects were associated with reduced iron accumulation and preservation of antioxidant capacity.

The coordinated changes in iron, lipid peroxidation, and antioxidant defense provide stronger evidence of ferroptosis-related alterations than any individual marker alone. However, these biochemical alterations alone do not establish ferroptosis as the sole form of cell death contributing to PS-MP-induced reproductive injury. Because ferroptosis shares several features with other forms of oxidative cellular injury, additional genetic or pharmacological approaches are needed to establish its specific contributions. In this context, the findings of the present study suggest that ferroptosis-related oxidative processes are associated with PS-MP-induced reproductive injury and are substantially attenuated by PC treatment. The SLC7A11/GPX4 pathway serves as an important endogenous defense against lipid peroxidation [29]. SLC7A11, a functional component of system Xc-, facilitates cystine uptake and supports GSH synthesis, whereas GPX4 uses GSH to reduce lipid hydroperoxides and protect membrane integrity [30]. Disruption of this system can increase cellular susceptibility to lipid peroxidation. In the present study, PS-MP exposure reduced SLC7A11 and GPX4 expression in testicular tissue, coinciding with increased iron accumulation, lipid peroxidation, and depletion of antioxidant defenses. This coordinated response suggests that impaired SLC7A11/GPX4 signaling may contribute to the inability of testicular cells to effectively control lipid peroxide accumulation following PS-MP exposure. Conversely, PC treatment restored SLC7A11 and GPX4 expression and improved GSH levels and other antioxidant parameters. These findings suggest that preserving SLC7A11/GPX4-mediated antioxidant defenses may contribute to the protective response induced by PC. The relationship between SLC7A11/GPX4 and the broader antioxidant network is particularly relevant to understanding PC-mediated protection. Rather than simply reducing oxidative damage, enhanced activity of the endogenous cystine-GSH-GPX4 system could increase the cellular capacity to eliminate lipid peroxides. This may explain why PC treatment was accompanied by improvements in multiple oxidative stress markers, rather than reduction in a single oxidative marker. Thus, the observed increases in SLC7A11 and GPX4 expression may provide a mechanistic link between the antioxidant effects of PC and the attenuation of ferroptosis-related changes observed in PS-MP-exposed mice.

NRF2 is an important regulator of cellular antioxidant defenses and has been implicated in the regulation of ferroptosis-related responses [47]. The NRF2 pathway also plays an important role in maintaining redox homeostasis in reproductive tissues [48]. In the present study, PS-MP exposure increased KEAP1 expression and decreased NRF2 expression, along with reduced expression of SLC7A11 and GPX4. In contrast, PC treatment decreased KEAP1 expression and increased NRF2 expression, accompanied by increased SLC7A11 and GPX4 expression. These findings suggest that alterations in NRF2 signaling are associated with changes in the downstream SLC7A11/GPX4 antioxidant defense system following PS-MP exposure. Inhibition of NRF2 by ML385 weakened the PC-associated recovery of SLC7A11 and GPX4 and reduced the protective response to PS-MP exposure. These findings support the involvement of NRF2 in regulating the antioxidant response associated with PC treatment. However, the present results did not establish NRF2 as a direct molecular target of PC. Changes in NRF2 signaling may also reflect a broader cellular response to changes in cellular redox status. Accordingly, the ML385 results are best interpreted as pharmacological evidence supporting the involvement of NRF2, rather than definitive evidence that PC directly activates NRF2.

Cellular experiments further strengthened the mechanistic interpretation of the in vivo findings. Leydig cells are responsible for testosterone synthesis and are, therefore, highly relevant to the reduction in serum testosterone observed in PS-MP-exposed mice. In PS-MP-treated TM3 cells, PC reduced Fe^2+^ accumulation and lipid peroxidation and improved mitochondrial function. These findings indicate that PC can modulate cellular responses to PS-MPs in the Leydig cell model. Changes in mitochondrial membrane potential and MPTP opening further suggest that PS-MP-induced oxidative stress is associated with mitochondrial dysfunction. Mitochondrial dysfunction may also contribute to impaired Leydig cell function because mitochondria participate in energy metabolism and steroidogenesis [49]. However, mitochondrial alterations are not specific to ferroptosis and may occur in response to multiple forms of cellular stress. Therefore, these results are best interpreted as complementary evidence of cellular injury and functional impairment rather than as independent ferroptosis-specific markers.

The role of SLC7A11-related signaling was further examined using Erastin. Erastin treatment attenuated the protective effects of PC in PS-MP-treated TM3 cells and was accompanied by increased Fe^2+^ accumulation and lipid peroxidation, together with worsening mitochondrial functional parameters. The associated changes in SLC7A11 and GPX4 expression further suggested that disruption of the Xc-dependent antioxidant defense system may compromise the protective response to PC. These findings were consistent with the in vivo observation that PC restored SLC7A11 and GPX4 expression. Taken together, the Erastin and ML385 experiments provide complementary pharmacological evidence implicating two components of the proposed regulatory axis: SLC7A11-related ferroptosis defense and NRF2-associated antioxidant regulation. Attenuation of PC-mediated protection following inhibition at either level supports the involvement of the NRF2/SLC7A11/GPX4 axis in the observed cellular response.

Molecular docking provided additional structural insights into the potential interactions of PC with NRF2 and SLC7A11. PC exhibited predicted binding energies of −10.1 kcal/mol with NRF2 and −8.6 kcal/mol with SLC7A11, indicating favorable predicted interactions in the docking models. These results provide a structural basis for the possibility that PC may interact with components of the NRF2/SLC7A11 regulatory system. However, docking scores cannot establish direct physical binding, target specificity, or the sequence of molecular events occurring in the cells. Therefore, the docking results should be considered supportive, but not definitive, mechanistic evidence. In particular, the more favorable predicted binding energy for NRF2 should not be interpreted as evidence that NRF2 is the primary target of PC. Direct binding assays, target-engagement studies, or genetic approaches are required to determine whether PC directly interacts with NRF2 or SLC7A11 and to clarify their respective roles in the protective response.

An important strength of the present study is the integration of reproductive phenotypes, biochemical indicators, molecular signaling, and pharmacological interventions across in vivo and in vitro models. Mouse experiments showed that PS-MP exposure was associated with impaired testosterone production, reduced sperm quality, and pathological changes in reproductive tissues, whereas PC ameliorated these reproductive outcomes. The accompanying changes in iron accumulation, lipid peroxidation, antioxidant capacity, and SLC7A11/GPX4 expression were consistent with ferroptosis-related oxidative injury in the testes. TM3 cell experiments further showed that PC modulated these cellular responses, while Erastin and ML385 weakened PC-mediated protection at distinct levels of the proposed pathway. Overall, findings across different experimental approaches are broadly consistent and support a model in which modulation of the NRF2-associated SLC7A11/GPX4 antioxidant defense may contribute to the PC-mediated protection against PS-MP-induced reproductive injury.

Several limitations should be acknowledged. The present study was performed using a mouse model and the TM3 cell line. Although these models provide useful evidence of reproductive and cellular responses to PS-MPs, validation in primary Leydig cells, additional reproductive cell types, and other animal models would strengthen the generalizability of our findings. Erastin and ML385 are pharmacological inhibitors that may exert off-target effects. Therefore, genetic approaches, such as NRF2 or SLC7A11 knockdown or knockout, would provide stronger evidence for the specificity and causal involvement of this pathway. In addition, molecular docking provides computational predictions rather than direct evidence of molecular binding; therefore, experimental validation using direct binding or target-engagement assays is needed. Although the present findings support an important role for ferroptosis-related oxidative injury, the contribution of other regulated cell death pathways, including apoptosis- and autophagy-associated processes, cannot be excluded. The long-term protective effects, optimal dosage, and safety of PC for translational applications are yet to be determined. Future studies incorporating genetic models, direct target validation, and long-term intervention studies will help clarify the molecular mechanisms underlying PC-mediated protection and its potential therapeutic relevance for MP-associated reproductive injuries.

## 5. Conclusions

The present study demonstrates that PS-MP exposure is associated with substantial impairment of male reproductive function, accompanied by iron accumulation, lipid peroxidation, depletion of antioxidant defenses, and suppression of the SLC7A11/GPX4 system. PC treatment alleviated these reproductive and biochemical abnormalities in vivo and improved the cellular response to PS-MP exposure in TM3 cells. Inhibition of NRF2 or SLC7A11-related signaling weakened the protective effects of PC, supporting the involvement of the NRF2/SLC7A11/GPX4 axis in this process. Overall, these findings suggest that modulation of NRF2-associated ferroptosis-related oxidative responses contributes to the protective effects of PC against PS-MP-induced male reproductive injury and provide a potential mechanistic basis for further investigation of natural antioxidants in microplastic-associated reproductive injury.

## Data Availability

The original contributions presented in this study are included in the article. Further inquiries can be directed to the corresponding authors.

## Supplementary Materials

The following supporting information can be downloaded at https://www.mdpi.com/article/10.3390/ani16213382/s1, Table S1: Primer sequences; Table S2: Antibody sources and dilution ratios; Figure S1: Western blot raw images of Figure 3; Figure S2: Western blot raw images of Figure 7; Figure S3: Western blot raw images of Figure 8.
