# Supplementary material for: Procyanidin Protects Against Polystyrene Microplastic-Induced Male Reproductive Injury Through the NRF2/SLC7A11/GPX4 Pathway
Source: Animals (Basel). 2026 Oct 27;16(21):3382. doi: 10.3390/ani16213382 (PMC13762345; doi:10.3390/ani16213382)

Table S1 Primer sequences

| Gene | Forward primer (5'→3') | Reverse primer (5'→3') |
| --- | --- | --- |
| FTH1 | TTGTATGCCTCCTACGTCTATC | CAGTCATCACGGTCTGGTTT |
| GSH-PX | GAAGTGCGAAGTGAATGGTG | GCAGGGTTTCTATGTCAGGTT |
| PTGS2 | TGAGTACCGCAAACGCTTCT | TGGGAGGCACTTGCATTGAT |
| TFRC | TGGAGACTACTTCCGTGCTA | CTGGTTTCTGAAGAGGGTTT |
| GPX4 | GGAGCCCATTCCTGAACCTTT | GGTGACGATGCACACGAAAC |
| SLC7A11 | ACCGTCATCGGATCAGGCAT | GTTCCACCCAGACTCGAACA |
| NRF2 | CAGAGTGATGGTTGCCCACT | CACACACTTTCTGCGTGCTC |
| KEAP1 | ACCAGATTGACAGCGTGGTT | CATACAGCAAGCGGTTGAGC |
| GAPDH | GCAGTGGCAAAGTGGAGATT | CCCAATACGGCCAAATCCGT |

TableS2. Antibody sources and dilution ratios

| **Antibody Name** | **Source** | **Dilution ratio** |
|---|---|---|
| FTH1 | Wanleibio | 1:1000 |
| GPX4 | Abmart | 1:1000 |
| SLC7A11 | Abmart | 1:1000 |
| KEAP1 | Abmart | 1:1000 |
| TFRC | Wanleibio | 1:1000 |
| NRF2 | Proteintech | 1:3000 |
| GAPDH | Proteintech | 1:2000 |

Figure S1: Western blot raw images of Figure 3

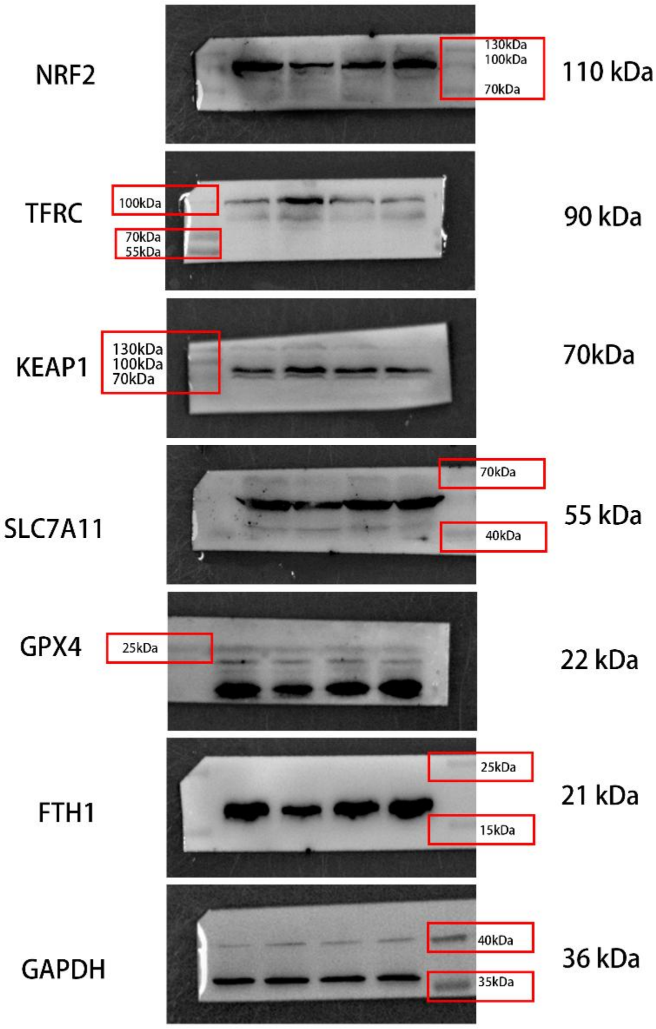

Figure S2: Western blot raw images of Figure 7

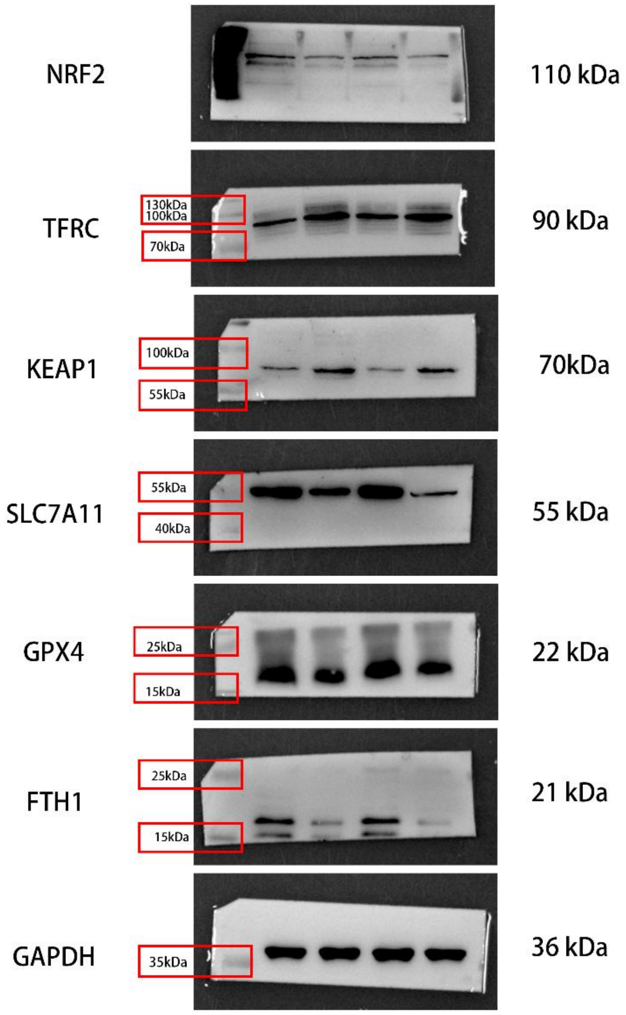

Figure S3: Western blot raw images of Figure 8

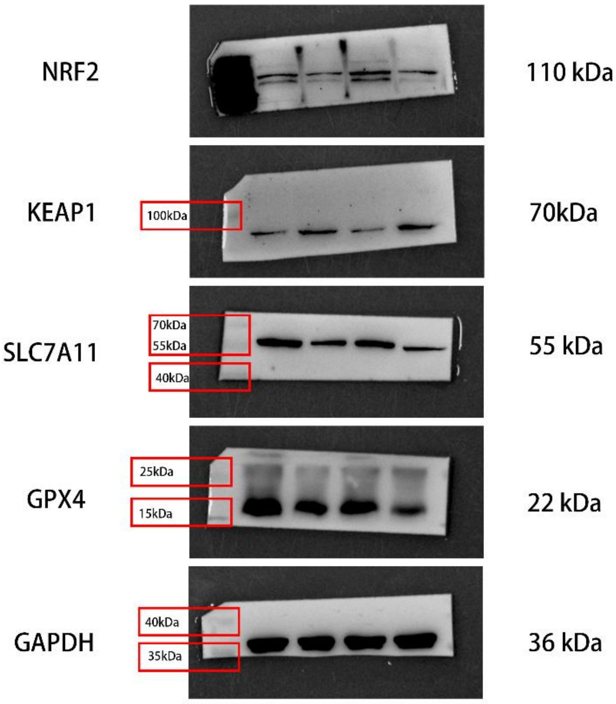

Supplement: Supplementary file 1 [file animals-16-03382-s001.zip › animals-4603951-supplementary.pdf]
